# A Review on Electrical Conductivity of Nanoparticle-Enhanced Fluids

**DOI:** 10.3390/nano9111592

**Published:** 2019-11-09

**Authors:** Alina Adriana Minea

**Affiliations:** Faculty of Materials Science and Engineering, Technical University “Gheorghe Asachi” from Iasi, Bd. D. Mangeron no.63, Iasi 700050, Romania; aminea@tuiasi.ro; Tel.: +40-723-455071

**Keywords:** nanofluids, electric conductivity, heat transfer

## Abstract

This review discusses exclusively the recent research on electrical conductivity of nanofluids, correlations and mechanisms and aims to make an important step to fully understand the nanofluids behavior. Research on nanoparticle-enhanced fluids’ electrical conductivity is at its beginning at this moment and the augmentation mechanisms are not fully understood. Basically, the mechanisms for increasing the electrical conductivity are described as electric double layer influence and increased particles’ conductance. Another idea that has resulted from this review is that the stability of nanofluids can be described with the help of electrical conductivity tests, but more coordinated research is needed. The purpose of this article is not only to describe the aforementioned studies, but also to fully understand nanofluids’ behavior, and to assess and relate several experimental results on electrical conductivity. Concluding, this analysis has shown that a lot of research work is needed in the field of nanofluids’ electrical characterization and specific applications.

## 1. Introduction

Heat-exchange processes are of major importance for almost all industrial processes, and thus their efficiency is of paramount significance. In the last few decades, a new class of heat-transfer fluids was developed and intensively studied, namely nanoparticle-enhanced fluids. This new class of fluids actually consists of regular heat-transfer fluids enhanced with solid nanoparticles, generally termed as nanofluids. As base fluids, both conventional and non-conventional fluids were considered, and a few examples are: water, ethylene glycol, oils, ionic liquids, basic lubricants and also molten salts. On the other hand, nanoparticles consist of metals, oxides, carbon nanotubes, graphene and several composites. The combination of these two phases (i.e., liquid and solid nanoparticles) raised a lot of interest in the published literature also due to their intrinsic applications in heat exchangers used for different industries as: automobile (i.e., car radiator), coolers, radiators, refrigerators, in the oil and gas industries (i.e., cooling and preheating of fluids), solar collectors, electronic industries, aeronautics etc [1,2].

Nevertheless, unlike the properties of regular mixtures that can be predicted very easily by averaging the properties of the pure phases, the thermophysical properties of nanofluids do not respect this rule, as was outlined intensively in the open literature (see for example [1,2,3,4,5,6,7,8,9,10,11,12]).

If the electrical conductivity is considered, this author believes that this is a less studied property, even if it is of tremendous relevance for several industrial applications. For example, commonly, fluids are poor conductors of electricity while several liquids (as for example: mercury, sea water, molten metals, electrolytes) are good conductors. In the last few years, an abundant consideration was received by the study of conducting fluids, especially because of their numerous applications in engineering, as for example: plasma jet, controlled thermo-nuclear reactor, shock tubes, pumps, magneto hydrodynamic generators [13,14,15,16]. Many gaps still exist in the science describing the flow of electrically conducting fluids and such gaps are most frequent with regard to magneto hydrodynamic (MHD) subjects like flows of inhomogeneous and multiphase fluids (i.e., nanofluids) and turbulent flows [13].

On the other hand, as one of the most relevant applications of nanofluids is electronic cooling, the increased electrical conductivity over the base fluid constitutes a major advantage [15], especially when it is coupled with higher thermal conductivity. Consequently, Pordanjani et al. [1] recommended in their comprehensive review the use of nanoparticles in heat exchangers under the influence of electrical fields, and thus the investigation of electrical behavior of nanofluids is of major importance.

Concluding, this review’s scope is to summarize research on electrical conductivity that is a very important property, especially for applications in mineral processing systems, fuel cells, electric field heat transfer applications etc. (see Pordanjani et al. [1] for example). 

As far as this author is aware, a complex review on electrical conductivity is not available at this moment and interest in measuring this property has been relatively limited so far. The purpose of this article is not only to describe the available experimental and theoretical studies, but also to gain a better awareness of the nanofluids behavior while evaluating and relating recent results on electrical conductivity measurements. Thus, this review’s starting point will be to summarize the theoretical models available for electrical conductivity, followed by the experimental research performed by now in regard to the influence of base fluid type, nanoparticle selection and temperature on electrical conductivity variation.

## 2. Theoretical Models for Electrical Conductivity

Banisi et al. [17] performed a very good review on electrical conductivity of dispersions, summarizing some models that are used for the estimation of electrical properties. They described the available equations and provided a distinct consideration of models which consider volume concentration, shape and size distribution of the solid dispersed phase, specifically the models of Maxwell [18], Bruggeman [19] and Fricke [20], discussing the limitations of each approach. In this sense, a short outline of theoretical models will be the start point of this review and a few aspects will be discussed further in correlation with experimental results.

The Maxwell model [18] is considered to be applicable mostly for low concentrations of spherical nanoparticles and estimates the electrical conductivity of the nanofluid as a function of electrical conductivity of nanoparticles and of the base fluid [17,18]:

(1) $$\frac{\sigma_{nf}}{\sigma_{bf}}=1+\frac{\dot{3\left(\left(\frac{\sigma_{p}}{\sigma_{bf}}\right)-1\right)\varphi }}{\left(\frac{\sigma_{p}}{\sigma_{bf}}\right)+2-\left(\left(\frac{\sigma_{p}}{\sigma_{bf}}\right)-1\right)\varphi }$$

Cruz et al [21] suggested, based on the classical model of Maxwell, other equations as:

(2) $$i)\;\frac{\sigma_{nf}}{\sigma_{bf}}=1-\frac{3}{2}\varphi ,\;\mathrm{for}\;\sigma_{p}\;«\;\sigma_{\mathrm{bf}}\;(\mathrm{insulating}\;\mathrm{particles})\;\mathrm{ii})\;\frac{\sigma_{nf}}{\sigma_{bf}}=1,\;\mathrm{for}\;\sigma_{p}\;=\;\sigma_{\mathrm{bf}}\;(\mathrm{equal}\;\mathrm{conductivity})\;\mathrm{iii})\;\frac{\sigma_{nf}}{\sigma_{bf}}=1+3\varphi ,\;\mathrm{for}\;\sigma_{p}\;»\;\sigma_{\mathrm{bf}}\;(\mathrm{highly}\;\mathrm{conducting}\;\mathrm{particles})$$

Cases (i)–(iii) display the theoretical effect, as predicted by the Maxwell’s model, of the particle volume fraction on the relative electrical conductivity.

On the other hand, the Bruggeman model [19] is:

(3) $$1-\varphi =\frac{\sigma_{p}-\sigma_{nf}}{\sigma_{p}-\sigma_{bf}}{\left(\frac{\sigma_{bf}}{\sigma_{nf}}\right)}^{1/3}$$

Mutually, Maxwell and Bruggeman equations can be used for electrical conductivity estimation, but their applicability is argued intensively in the open literature as will be discussed in the next sections.

Alternatively, Fricke [20] considered the general case of a suspension of homogeneous ellipsoids, and his model actually reduces to the Maxwell model for spherical particles. That is the reason why this model was not considered as a base for comparison in the open literature, where almost all considered nanoparticles are spherical (i.e., with some exceptions, as different kinds of carbon nanotubes).

## 3. Literature Overview

The importance of electrical conductivity estimation was more intensively outlined about 3–4 years ago when the research on this topic clearly increased, thus not much experimental work was identified in the archived literature. This review is fully dedicated to electrical conductivity of nanofluids, being initially to try to summarize and discuss experimental outcomes on this property. Accordingly, Table 1 outlines the most recent and relevant researches related to electrical conductivity, containing details about the base fluid, nanoparticles type and also about the equipment used for experiments. Table 1 can offer a very good start point for future research, summarizing some preoccupations and contains all the available data, as far as this author is aware.

Another point to be raised is the electrical conductivity of the base fluid, illustrated in Table 2, as measured by several authors. This is of high relevance since all the research groups are discussing the electrical conductivity enhancement, as a comparison between their results and the base fluid experimental outcome. One can easily notice the scattered results, which may depend on the purity of each base fluid as well as the equipment (type and its calibration). Nevertheless, while a conducting fluid generally has an electrical conductivity larger than 10 µS/cm [16], each author compared their research on nanoparticle addition to their base fluid measured data. If results from Table 1 and Table 2 are compared, it may easily be noticed that nanoparticle addition changes the fluid behavior, transforming, in most cases, a non-conducting fluid in a conducting one.

Below, the discussion will continue with details about performed experiments, categorized by the base fluid. Nanofluids with ethylene glycol (EG), water and different mixtures EG–W as the base fluid received an increase attention in the archived literature, while other base fluid definitely needs more research.

### 3.1. Nanofluids with Ethylene Glycol (EG) as Base Fluid

Shirazi et al. [22] synthesized and studied the thermo-electrical behavior of the nitrogen doped activated carbon/grapheme (NACG) hybrid with high nitrogen content from carbon derived from EFB pulp and GO. The electrical conductivity of three samples of EG-based nanofluids with different concentrations (i.e., 0.02–0.06%) was measured in the range of 20–45 °C and an increase was noticed while the samples’ concentration increased, reaching 12,000% enhancement in electrical conductivity at 30 °C. The equipment used for this study was a conductivity meter (AB200, Fisher scientific).

Mohamed [23] performed a theoretical study, using an artificial neural network (ANN) model, of the electrical properties of two nanofluids based on EG with MgO and Si-TiO nanoparticles. Electrical conductivity was simulated using the ANN model in regard to both nanoparticle concentration and temperature influence. The simulation involved several experimental outcomes from the literature and the result was a non-linear equation describing the electrical behavior of the nanofluids.

Akilu et al. [24] reports data on electrical conductivity of several ethylene glycol and propylene glycol based β-SiCnanofluids. The electrical conductivity was measured using a portable conductivity meter with inbuilt thermistor for automatic temperature compensation. The dependency of β-SiC NFs electrical conductivity on temperature was approximated by the authors with the help of an Arrhenius-type equation, while the variation with volume concentration was found to be linear. Moreover, the results were found not to be in line with the Maxwell model, as also other authors noticed (see for example 45–48). The overall conclusion was that the base fluid viscosity, ionic strengths, and the electrical double layer (EDL) interaction influences greatly the electrical conductivity of the nanofluid.

Baby and Ramaprabhu [26] studied the electrical conductivity of an EG nanofluid with graphene and an enhancement of about 220% was noticed.

Robust studies on electrical conductivity of EG based nanofluids were performed by the group from Zyla and Fall [25,27,28,29,30,31]. For example, Fal et al. [25] investigated the indium oxide-EG nanofluids with MultiLine 363 equipment and found an increase in electrical conductivity of nanofluids both with concentration and temperature. The maximum growth in electrical conductivity of In_2_O_3_-EG nanofluids was detected for 0.0081% concentration at 333.15 K, namely 27,300%.

Zyla et al. [27] prepared two types of EG based nanodiamonds nanofluids and their results showed a nonlinear progression in electrical conductivity with volume fraction increase and a correlation was developed as:

(4) $$\frac{\sigma_{nf}}{\sigma_{bf}}=1+3734\varphi -25.65\varphi^{2}$$

The augmentation in electrical conductivity was attributed to EDL and conducting paths created by the nanoparticles inside of the EG liquid.

Zyla and Fal [28] performed experiments on aluminum nitride dispersed in EG and noticed an increase in electrical conductivity with the upsurge in nanoparticles concentration and a new correlation was proposed as:

(5) $$\frac{\sigma_{nf}}{\sigma_{bf}}=1+6950.56\varphi$$

Moreover, authors agreed that the Maxwell model does not fit the experimental data and the real augmentation is much higher (up to 600 times if compared to base fluid) than that predicted by the Maxwell model.

A linear increase of electrical conductivity with nanoparticle concentration was found also for the transparent suspensions of silicon dioxide (SiO_2_) nanoparticles in EG at 298.15 K and the correlation is [29]:

(6) $$\frac{\sigma_{nf}}{\sigma_{bf}}=1+21.03\varphi$$

This correlation was obtained by fitting the experimental data with standard error of 0.72.

Additionally, Zyla and Fal [29] performed a thermo-electrical conductivity (TEC) analysis in terms of electrical and thermal conductivity and concluded that there are no benefits from using this type of nanofluid in a heat-transfer processes, since TEC varies from 4 to 6, in dependence with the volume fraction. 

Plus, the same group [30,31] dispersed silicon nitride (Si_3_N_4_) in ethylene glycol using a two-step method and measured electrical conductivity, while proposing a polynomial regression equation due to inconsistencies with the Maxwell law:

(7) $$\frac{\sigma_{nf}}{\sigma_{bf}}=1+78609\varphi -294573\varphi^{2}$$

Zyla et al. [31] explained this high growth in electrical conductivity by the combined effect of some causes, as: the nanoparticles concentration, the physical and chemical properties of the nanoparticles combined with the base fluid influence. All these factors were producing a pronounced EDL around nanoparticles and conduction paths.

### 3.2. Nanofluids with Water as Base Fluid

Water as the base for nanofluids preparation received increased attention from different research groups, mainly because of its large areas of applications. Furthermore, several outcomes on electrical conductivity of water-based nanofluids are discussed in this section.

Zawrah et al. [32] prepared alumina–water nanofluids with SDBS (Sodium dodecyl benzenesulfonate) as surfactant and measured their electrical conductivity for different concentrations. The highest value of electrical conductivity, 2370 µS/cm, was logged for 0.2% concentration at a temperature of 25.9 °C. Their experimental observations showed that the electrical conductivity increases with nanoparticle addition until 0.2% and decreases when nanoparticles concentration increases (i.e., for 0.5% and 0.75%). The explanations behind this variation in electrical conductivity lay with the EDL formation phenomenon and the electric charges development due to the fact that water is a polar liquid. More precisely, ions of charge opposite to that of the particle surface are attracted, causing the development of a charged diffuse layer surrounding the particle. This layer, known as the electrical double layer, is commonly characterized by the Debye length parameter. Authors declared that the actual enhancement mechanism is based on improving the conduction in the suspension due to surface charge and EDL formation. Plus, the increase in electrical conductivity was attributed to a better suspension stability. On the other hand, the decrease of electrical conductivity for 0.5 and 0.75 vol.% was explained due to the fact that charges available for the formation of EDL are insufficient for particles and the electrostatic attraction force is transformed into a repulsion force between nanoparticles in the nanofluid. These results are not in line with the other communicated ones and the explanations behind this phenomenon need further insight.

Bagheli et al. [33] experimentally studied a nanofluid based on water and iron oxide (with surfactant) and found a considerable enhancement of electrical conductivity with the upsurge in concentration and temperature. Authors also proposed a model that was able to explain the mechanism of Fe_3_O_4_ nanofluid electrical conductivity, especially at low concentrations. The proposed model is actually a verification of Shen et al. [58] model, equation that describes the electrical conductivity of nanofluids based on the Maxwell model (see Equation (1)) but also takes into account both the Brownian motion of particles and electrophoresis.

Shen et al. [58] improved the Maxwell model taking into account the conductivity due to electrophoretic mobility and Brownian motion and is written as:

(8) $$\sigma =\sigma_{M}+\sigma_{E}+\sigma_{B}$$

where $\sigma_{M}$ and $\sigma_{B}$ refers to the electrical conductivity calculated with the Maxwell model and due to Brownian motion, respectively. The electrophoretic mobility ($\sigma_{E}$) writes:

(9) $$\sigma_{E}=\frac{2\varphi \varepsilon_{r}^{2}\varepsilon_{0}^{2}U_{0}^{2}}{\eta r^{2}}$$

where *ε_r_*, *ε_0_*, *U_0_*, *η* and *r* are the dielectric constant of base fluid, dielectric constant of vacuum, zeta potential of nanoparticles, viscosity of the nanofluid and the radius of nanoparticles.

The term related to Brownian motion is:

(10) $$\sigma_{B}=\frac{3\varphi \varepsilon_{r}^{}\varepsilon_{0}^{}U_{0}^{}\left(\frac{RT}{L}\cdot \frac{1}{3\pi \eta }\right)}{r^{3/2}}$$

where *R*, *T* and *L* are the thermodynamic constant, temperature and Avogadro constant, respectively.

The electrical conductivity of water-based nanofluids with copper oxide (12 nm) was studied by Coelho et al. [34]. The authors prepared various nanofluids (in concentrations up to 2%) and undertook experiments at different temperatures, in the range of 298.15 to 348.15 K, using a conductivity meter type EC-Meter GLP 31 from CRISON. Their results are in line with the open literature and show an enhancement of electrical conductivity with the increase both in temperature and volume concentration. Their explanation for this augmentation relies on the high value of nanoparticles’ electrical conductivity. Moreover, the experimental data were found to be in line with the Hill equation [59]:

(11) $$\sigma =\frac{\varphi_{CuO}^{n}K_{0}^{}}{1+\varphi_{CuO}^{n}K_{0}^{}}$$

As an explanation, Hill equation has two fitting parameters, *K*_0_ and *n*, and was used in different branches of science to quantitatively describe the degree of cooperation in different kinetic processes [60].

Anu and Hemalatha [34] studied the electrical conductivity of undoped and zinc-doped cobalt ferrite nanoparticles suspended in water and compared the results with Maxwell [18] and Shen’s [58] models. The comparison revealed that the Maxwell model is not suitable (i.e., it under predicts the experimental values) while Shen’s equation can describe the experimental outcomes. These authors’ outcomes are in line with the observations from Bagheli et al. [33].

Shoghl et al. [36] performed an experimental study using a JENWAY 4520 conductivity meter on several water-based nanofluids with alumina, CuO, MgO, CNT, titania, ZnO and found several linear correlations, while the increase is explained through EDL and ionic conduction. The maximum upsurge was noticed for ZnO-water nanofluids, but no in-depth explanation behind this phenomenon was provided.

Heyhat and Irannezhad [37] investigated Ag, SiC, and Graphene oxide (GO) water-based nanofluids using a AZ86505 benchtop multi-meter (AZ Instrument Corp.). The results are in line with literature and in almost all cases the electrical conductivities of nanofluids is linearly increasing with temperature and concentration.

Nurdin and Satriananda [38] investigated the Fe_2_O_3_–water nanofluids with an Eutech instrument PC 2700 in the range of 0.5–2.5% volume concentrations of nanoparticles. The highest value of electrical conductivity (14.65 mS/cm) was attained at 2.5% concentration and 60 °C and attributed the increase in electrical conductivity to the complicated dependence on the electrical double layer.

Mashali et al. [39] studied the nanodiamond water based nanofluids in three concentrations up to 0.25 wt.% and performed a comparison with existing literature. Outcomes concluded that adding nanodiamond obtains the minimum electrical conductivity if compared with other nanoparticle types.

Baby and Ramaprabhu [26] scanned the electrical conductivity of the nanofluid with graphene/W and an enhancement of about 1400% was noticed.

Zakaria et al. [41] investigated the properties of 0.1%, 0.3% and 0.5% Al_2_O_3_ nanoparticles dispersed in water and found an increase of about 5.5 times if compared with pure water. The explanations are based on the fact that the 0.5% Al_2_O_3_ nanofluids in water picks up most ions from the stack as there is an addition of 29.0 µS/cm.

Alumina–water nanofluids were also studied by Selimefendigil and Öztop [43] who performed a numerical analysis in mixed convection in a lid-driven trapezoidal cavity using different equations for electrical conductivity and concluded that the disagreement between diverse electrical conductivity models becomes higher for upper values of Richardson number. Ganguly et al. [44] noticed a considerable augmentation of electrical conductivity of alumina–water nanofluids with temperature and volume fraction. The variation was found to be linear in terms of both temperature and volume fraction and a new correlation was proposed as:

(12) $$\frac{\sigma_{nf}-\sigma_{bf}}{\sigma_{bf}}=3679.049\varphi +1.085799T-43.6384$$

Minea and Luciu [45] and Minea [46] also examined alumina–water nanofluid and noticed an increase of electrical conductivity both with temperature and volume concentration. A new correlation was proposed as:

*σ_nf_* = 176.69 + 588.41*φ* − 13.64*t* − 86.31*φ*^2^ + 0.36*t*^2^ + 1.07*tφ* + 11.06*φ*^3^ − 0.003*t*^3^ + 0.18*t*^2^*φ* − 1.01*tφ*^2^ (13)

where *t* refers to temperature in °C and *φ* is the volume concentration.

Further findings can be summarized as: at room temperature an increase of 379.6% in effective electrical conductivity of nanofluid was detected for 4% alumina and a linear increase of electrical conductivity with temperature was noticed.

Sundar et al. [47] performed experiments on ND (nanodiamond)–Ni nanocomposite + water nanofluid and found a 1339.81% enhancement in electrical conductivity at 24 °C. Plus, a disagreement was noticed between experimental results and conventional models (i.e., Maxwell and Bruggeman), as many authors found.

Chereches and Minea [48] performed measurements of electrical conductivity of some simple and hybrid nanofluids based on water and different oxides (i.e., alumina, titania, silica) and few correlations were proposed based on volume fraction and temperature variation. For example:

for silica water nanofluids:

*σ_nf_* = −103.47 + 315.14*φ* + 17.23*φ*^2^ + 4.45*T* (14)

for titania water nanofluids:

*σ_nf_* = 491.56 + 104.67*ϕ* + 71.37 *ϕ*^2^ + 4.19*T* (15)

The overall experimental results for all simple and hybrid nanofluids were found to be mostly in line with similar research on different water-oxides nanofluids (see for example [26,41,43,44]).

### 3.3. Nanofluids with Water-Ethylene Glycol Mixture as Base Fluid

Islam et al. [49] investigated, both theoretically and experimentally, the electrical conductivities of 50/50 water-EG based TiO_2_ nanofluids with low nanoparticle concentrations (i.e., from 0.05 to 0.5 vol.%). The experimental results point out once again that the Maxwell model is not able to correctly predict the electrical conductivity of nanofluids. The experimental values were increasing with both temperature and concentration and this change was attributed to the EDL properties.

Islam and Shabani [50] used an IntelliCALTM CDC401 to measure the electrical conductivity for titania–water + EG nanofluid and the results showed an increase with both temperature and concentration. The base fluid was a water–EG mixture in equal proportion while the nanoparticles volume fraction was varied in the range 0.05%–0.5%. The authors proposed a correlation as:

(16) $$\frac{\sigma_{nf}}{\sigma_{bf}}=11.214+2.626ln\varphi +0.2371lnT$$

Other oxide nanoparticles, Al_2_O_3_, were investigated by Zakaria et al. [41] in concentrations of 0.1, 0.3 and 0.5% dispersed in water–EG mixtures of 60:40. Results clearly depicted that the change in electrical conductivity is rather low due to some factors such as the oxidation of glycol and contamination from the bipolar plate.

Sarojini et al. [51] performed a large experiment on electrical conductivity of nanofluids containing either metallic or oxide nanoparticles (Cu, Al_2_O_3_, and CuO) with different low-volume fractions and particle sizes. Authors noticed that the electrical conductivity increases with increasing particle concentration and when particle size reduces. The outcomes of this complex study revealed that the Maxwell model under-predicts the experiment and the explanation stands on the EDL and the surface conductance of the particles.

Guo et al. [52] manufactured nanofluids with silicon oxide in an EG–water mixture and measured the electric conductivity with an electric conductometer (Jenco Instruments Inc., America). Authors found an enhancement in electrical properties (i.e., the electrical conductivity rises by about 10 times) due to EDL development while nanoparticles are added to the base EG–water mixture.

Graphene nanofluids received little attention if compares to the oxide based nanofluids [53,54], nevertheless, all studies indicate a large electrical conductivity increase (over 1000%), even at very low nanoparticle loading. Subsequently, Ijam et al. [53] experimentally investigated the electrical conductivity of graphene nanoparticles dispersed in a mixture of water–EG (mixing ration of 60:40) using an Orion™ VERSA STAR™ pH/Conductivity Multiparameter Benchtop Meter. The results showed that the electrical conductivity rapidly increased with loading of GONs (graphene oxide nanosheets) until 0.07 wt.%. The experimental results were fitted with a linear equation and different correlations were proposed for each nanofluid electrical conductivity variation with temperature. More precisely, at room temperature the maximum improvement in electrical conductivity was 1664% at a weight fraction of 0.10%. The authors’ explanation for the electrical conductivity increase relied on the surface charge of the GONs that strengthen the EDL together with the ion cloud, thus actively contributing to the enhancement in conduction mechanisms through the dispersion.

Kole and Dey [54] studied electrical conductivity of W–EG having functionalized graphene nanosheets (f-HEG) and results showed that the electrical conductivity enhanced to a percentage up to 8620% if related to the base fluid.

### 3.4. Nanofluids Based on Other Liquids

Research on the electrical conductivity of nanofluids based on other liquids is very limited and the results are inconsistent. This can be explained easily by the base fluid electric properties and its synergy with different kinds of nanoparticles. Furthermore, several studies were found in the archived literature, as further discussed in this section and outlined in Table 1.

#### 3.4.1. Bioglycol-Based Nanofluids

Khdher et al. [55] considered alumina–BG nanofluids of 0.1, 0.3, 0.5, 0.7, and 1 %vol. concentration in nanoparticles. The experimental results revealed that the electrical conductivity is increasing by both temperature and volume fraction. Plus, compared to base fluid, adding Al_2_O_3_ leads to a slight increase in electrical conductivity; for example, at 80 °C and 0.5% concentration, the value of electrical conductivity was 154 μS/cm. The explanation of this phenomenon was attributed to the configuration of surface charges by nanoparticle’s polarization effect once dispersed in a polar fluid.

Alumina nanoparticles (in low concentrations of up to 2%) were considered also by Abdolbaqi et al. [56], but this time the base fluid was a mixture of water and bio glycol (BG. Authors measured the electrical conductivity with a Cyberscan PC-10 and results showed a decrease while concentration increases, thus not following the base fluid behavior. More precisely, the effective electrical conductivity of BG–W in a 40:60 ratio decreased progressively from 620 to 472 µS/cm for volume concentrations of 0% and 2.0%, respectively.

#### 3.4.2. Oil-Based Nanofluids

Naddaf and Heris [57] used diesel oil as base fluid to prepare nanofluids with graphene and multi-wall carbon nanotubes (MWCNT) (using two kinds of surfactants: oleic acid and hexylamine) and noticed an increase in electrical conductivity while concentration increases. Also, nanofluids with functional nanomaterials have inferior electrical conductivity than non-functional nanomaterials and the explanation relies on EDL and surface charge. Nevertheless, the influence of surfactant was not sufficiently elaborated.

Huang et al. [61] used an eco-friendly vegetable liquid (i.e., refined, bleached and deodorized (RDB) oil) as base fluid to prepare nanofluids with fullerene nanoparticles and noticed an augmentation in the electrical properties. For example, the electrical resistivity increased by 23.3% at a concentration of 100 mg/L of fullerene nanoparticles.

Konakanchi et al. [62] dispersed different oxides (aluminum, silicon oxide and zinc oxide) into a mixture of propylene glycol and water. Their experimental results revealed that the nanofluid electrical conductivity upsurge with both temperature and nanoparticle concentration increase. The results obtained were in line with the literature and few empirical models were proposed by the authors.

## 4. Discussion on Experimental Results

The experimental results will be further compared for each base fluid, in order to be able to make a proper assessment and to draw a state of the art conclusion. Overall, the results are highly dependent on the nanoparticle type as well as on the manufacturing method and use of surfactants.

### 4.1. Nanoparticle Concentration Influence on Electrical Conductivity

**Figure 1:**
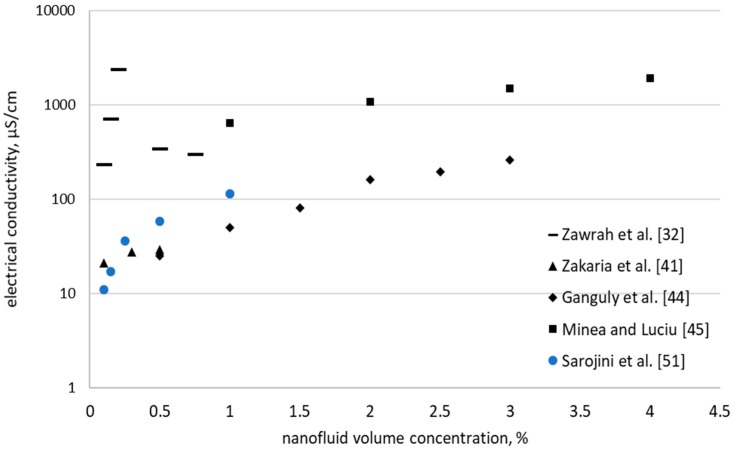
Electrical conductivity of alumina-water nanofluid.

Figure 1 depicts the results for alumina–water nanofluids and one can clearly notice the scattered data published in the open literature. As can see in Figure 1, the majority of experimental results indicated an increase of electrical conductivity with concentration increase. Nevertheless, some authors (see for example [32]) noticed an increase followed by a decrease when concentration rises. The explanation for this behavior was given by Zawrah et al. [32] who believed that the decrease in electrical conductivity appears due to the decrease of particle diameter (caused by the growth of surface area and the increased number of particles). Hence, because the number of particles increases, the charges available for the formation of EDL are insufficient and the electrostatic attraction force become a repulsion force among nanoparticles in alumina nanofluids. Anyhow, it is worth mentioning here that Zawrah et al. [32] used SDBS as surfactant while the other authors did not used surfactants.

Therefore, if we consider the results depicted in Figure 1 one can say that adding surfactants can greatly influence also the electrical behavior of nanofluids and the phenomenon that appears into the fluid needs further elaboration sustained by coordinated studies.

**Figure 2:**
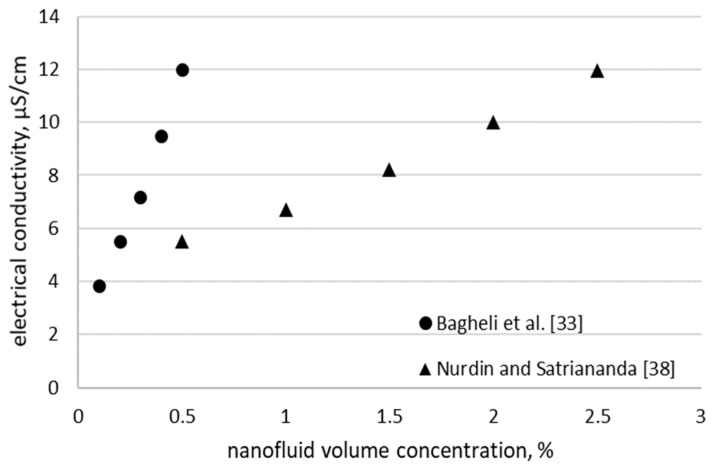
Electrical conductivity of iron oxide-water nanofluid.

The experimental results for the nanofluids with water and iron oxide are plotted in Figure 2, where two references were found. Bagheli et al. [33] obtained lower values, but used a surfactant (tetra methyl ammonium hydroxide). Anyhow, we cannot say exactly what is the influence of surfactant on electrical conductivity values if comparing results from Figure 1 and Figure 2, and thus more research is needed.

**Figure 3:**
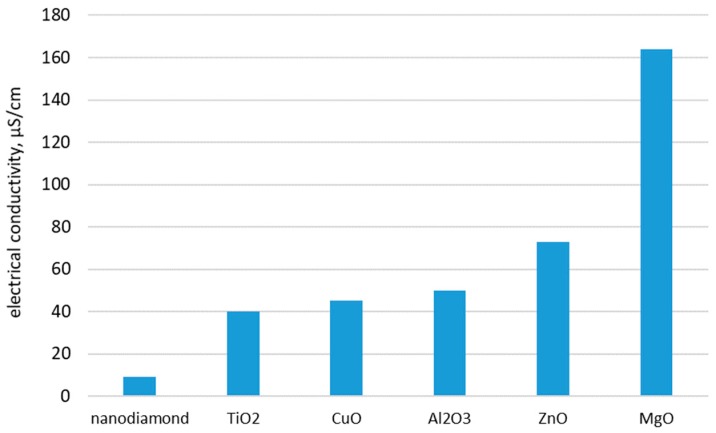
Comparison of electrical conductivity values for 0.01% nanofluid with different types of nanoparticles dispersed in water [36,39].

**Figure 4:**
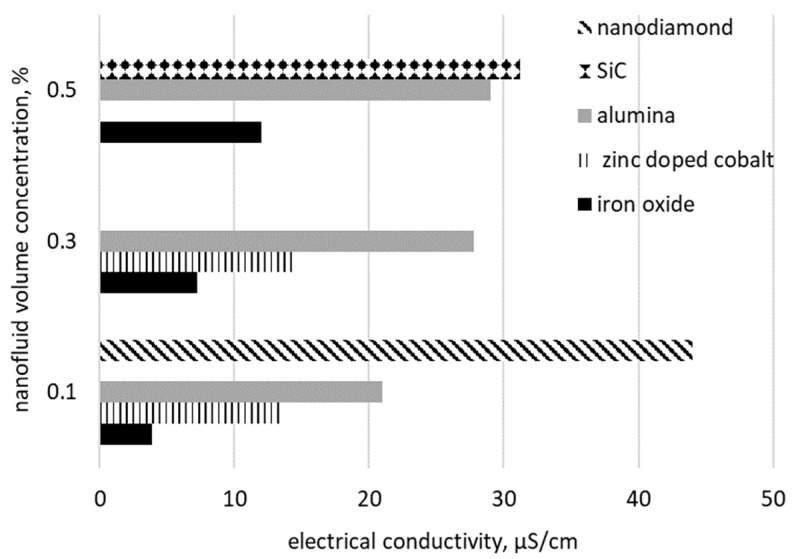
Electrical conductivity for different nanoparticle type [33,34,37,39,41].

Another interesting comparison can be attained based on the same volume concentration of nanoparticles dispersed in water, but different types (see Figure 3 and Figure 4). Figure 3 contains data for different kinds of nanoparticles of the same concentration in water (i.e., 0.01%) and it can be noted that the highest values were obtained for magnesium oxide while nanodiamond nanofluids has the lowest values. Alternatively, in Figure 4 it can be clearly noted that increasing the concentration to 0.1%, nanodiamond nanofluids have the highest values compared with data from other references.

If we look at data from Table 1, it can be noted that the experimental data for nanofluids with EG as base fluid are extremely scattered so a detailed conclusion of influencing factors cannot be ruled out. However, most of the results on EG nanofluids indicated a high augmentation of electrical conductivity if nanoparticles are added. Moreover, results on other base fluids, such as BG, oils or different mixtures are scarce, as was also affirmed before.

**Figure 5:**
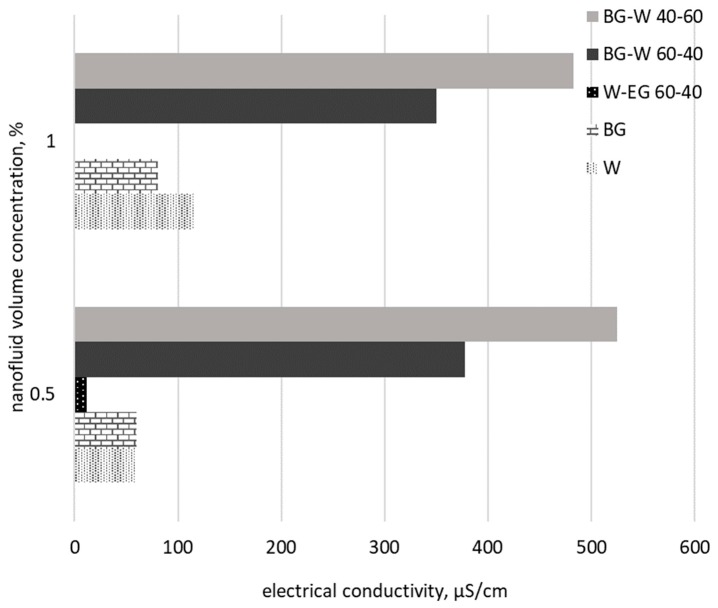
Comparison of data on alumina nanofluids with different base fluids [41,51,55,56].

Anyhow, based on reviewed data we can try a comparison of alumina nanoparticles suspended in different base fluids, as can be seen in Figure 5.

Looking at Figure 5, it can be said that using the same nanoparticle concentration the base fluid greatly influences the electrical conductivity mainly because of the EDL formation and the synergy between base fluid and nanoparticle. The highest values were obtained for a mixture between water and bio glycol and the minimum values are for EG as base fluid. This can be also due to the values registered for each base fluid electrical conductivity (see Table 2 and [41,51,55,56]). For example, water (with a medium electrical conductivity of 5.5 µS/cm [39,42]) has 10 times higher electrical conductivity if compared to EG, while BG electrical conductivity values reach 45 µS/cm [56].

Consequently, the increase of electrical conductivity with nanoparticle concentration was explained by most of the authors through several mechanisms that can be summarized as:

1. The complex processes that occur in the electrical double layer (EDL) formation and the interaction between the solid nanoparticles and the created EDL. 

2. The dependence on ionic concentrations and other physicochemical properties of the base fluid. 

3. Improvement in conduction mechanisms inside the suspension. 

4. Increased electrophoretic mobility of the nanoparticles (due to the nano dimensions order of particles) that subsequently strengthen the electrical conductivity of the nanofluid. 

5. The increase in nanoparticle concentration determine an increased availability of conducting path-ways in the nanofluid, which generate an escalation in the electrical conductivity. 

Anyhow, most of the researchers (see [19,20,21,22,23,24,25,26,27,28,29,30,31,32,33,34,35]) have explained the increase in electrical conductivity mainly by EDL formation; that is, the structure of charge accumulation and charge separation that always occurs at the interface when an electrode (in this case solid nanoparticles) is immersed into an electrolyte solution (i.e., the base fluid).

Basically, the EDL denotes two parallel layers of charge adjoining a solid body. The first layer, the surface charge (positive or negative), contains ions adsorbed because of chemical interfaces. The other layer contains ions attracted to the surface charge by means of the Coulomb force, electrically screening the first layer. This second layer is composed of free ions that are freely moving inside the base fluid under the effect of electric attraction and thermal motion.

### 4.2. Base Fluid Influence on Electrical Conductivity

Another interesting discussion can be made on the influence of the base fluid, especially in regard to its polarity. As is well known, oils are non-polar and water is a polar liquid, but EG is a symmetrical polar molecule, so it contains internal dipoles. In this idea, EG contains polar O-H groups but it has both polar and non-polar parts. Bio glycol is an aroma form of propylene glycol and has a wide range of polarity. On another hand, if these glycols are mixed with water, the polarity is influenced and the mixture can be considered polar.

Concluding, in regard to base fluid influence, the increase of electrical conductivity can be influenced by:

1. The dependence on ionic concentrations and other physicochemical properties of the base fluid. 

2. The use of surfactants. 

3. The polarity of the base liquid (i.e., water is a polar liquid but EG can be both polar and non-polar) that favours the creation of the electric charges on the nanoparticles surface. Ions of opposite charge to that of the particle surface are attracted, causing the advance of a charged diffuse layer adjacent to the nanoparticle. 

### 4.3. Temperature Influence on Electrical Conductivity

Studies on temperature variation influence over electrical conductivity are summarized in Table 3. The overall conclusion was that the temperature increase leads to a linear increase in the electrical conductivity [24,25,26,27,28,29,30,31,32,33,34,35,36,37,38,39,40,41,42,43,44,45,46,47,48,49,50,51,52,53,54,55,56,57] which is a logical phenomenon with major occurrence in the physics of suspensions. Some details are already presented in both Table 1 and Section 3 and a discussion will be undertaken below. From a state of the art review, it can clearly be noticed that most of the experimental studies on this topic concluded that temperature influence is not as major as concentration and the increase is linear. Anyhow, some exceptions were noted in the literature, as for example Shirazi et al. [22] stated that temperature influences the electrical conductivity but no pattern was noticed (i.e., actually very scattered data were registered) and Akilu et al. [24] found an Arrhenius type equation that better describe their experimental outcomes. On the other hand, some explanations for the low enhancement come from Sarojini et al. [51] who explained that the low enhancement in electrical conductivity at heating occurs due to the fact that aggregation is a time-dependent phenomena and the aggregation time is greatly reduced when temperature is increasing.

A drawback noticed, even if there are several studies of temperature influence on electrical conductivity, is the absence of correlations that can describe the heating influence. Even if temperature was considered as an important parameter, most of the equations are connecting both concentration and temperature effect, as can be seen from Equations (12)–(16).

### 4.4. Other Factors Influence on Electrical Conductivity

Other factors that may influence the properties of nanoparticle enhanced fluids can be outlined as the presence or absence of the surfactant, nanoparticle dimensions, and the method of preparation. Unfortunately, in regard to electrical conductivity, some systematic studies were not performed by now. Anyhow, some authors (see [36,51]) tried to shed some light on these aspects, as it will be outlined further.

The influence of surfactant was barely studied and no conclusion can be attained. This author found only two studies on this topic and the results are scattered.

Shoghl et al. [36] performed an interesting study involving several nanoparticles dispersed in water and water + SDS (Sodium dodecyl sulfate) at different concentrations. They noticed that pure water with SDS electrical conductivity increased with increasing surfactant concentration (i.e., 2 concentrations of SDS were considered: 0.01 and 0.02 wt.%). As for nanofluids with SDS, the influence of surfactant is correlated with the nanoparticle type. For example, for Al_2_O_3_, MgO, ZnO, TiO_2_ and CuO nanofluids, the addition of nanoparticles enhances both the electrical conductivity of the base fluid and of the base fluid + surfactant. However, a similar phenomenon was not noticed for the MWCNT nanofluids (i.e., with or without surfactant addition). For all nanofluids, excepting MWCNT, adding the surfactant leads to an increase in the electrical conductivity. Furthermore, these authors compared their results on carbon nanotubes and explanation on ionic conduction mechanism with that of Glover et al. [63] who used carbon nanotubes of up to 0.2 wt.% concentration dispersed in a 50:50 deionized water-EG solution. As an explanation, Glover et al. [63] experiments depicted a linear increase of electrical conductivity up to 13 times and their justification relies on ionic conductivity and functionality of the carbon nanotubes. Precisely, functional nanotubes would decrease the electrical conductivity compared to the un-functional prime nanotubes because they break the conjugated bond of the nanotube system. Nevertheless, these mechanisms need further elaboration and more experimental observations.

Sarojini et al. [51] investigated the effect of surfactant SDS (0.1 and 0.5 mM concentrations) and compared the results with no surfactant probes. It was found that the electrical conductivity enhancement in the presence of the SDS is higher at low concentration (up to 0.3%) and lower at higher concentrations (over 0.3%).

### 4.5. Electrical Conductivity—A Method for Stability Estimation?

There is a certain amount of research groups [32,44,50,61,64] that linked the electrical conductivity to nanofluids stability. Nevertheless, in this regard more research is needed to fully describe the phenomenon, even if some comments are present in the open literature. Tests have to be performed at a certain time distance and in correlation with zeta potential, for example. However, the explanation that stands at the base of this observation relies on the fact that when agglomeration occurs, clusters are formed and this decreases the surface potential of nanoparticles. The reduction in surface potential is clearly a signal of instability in a nanofluid and an unstable nanofluid will contain few electrically disconnected nanoparticles (charged ones) because of the reduced electrical potential. Moreover, the nanoparticles aggregation determines an increase of the particle size, a phenomenon with clear negative impact on Brownian motion and electrophoretic mobility of solid nanoparticles [51,60].

## 5. Conclusions

In this article, a complex review was performed on electrical conductivity results. Even if the other nanofluids’ properties received greater attention (see thermal conductivity, viscosity, specific heat), studies on electrical conductivity can also offer valuable information about these new fluids’ behaviour in different real-life applications. Some of the conclusions that can be derived from this state of the art review are summarized as follows:

1. Electrical conductivity, together with zeta potential, can be a good tool to evaluate the nanofluid stability; more precisely, the increase in electrical conductivity is attributed to a better suspension stability. Alternatively, a reduction in electrical conductivity suggest a poor stability and this property can be measured also at a certain time distance to check the long-term stability of a nanofluid.

2. Electrical conductivity depends on base liquid type and polarity.

3. Electrical conductivity is influenced by the addition of surfactants.

4. Electrical conductivity was found to increase with temperature upsurge; however, its variation with nanoparticle concentration is not fully described and understood, results being somewhat contradictory (i.e., most authors found an increase with concentration, but there are studies that contradict this hypothesis).

5. The increase in electrical conductivity was found to be mainly determined by three causes: surface conductance of nanoparticles; electrical double layer development, liquid polarity.

6. The Maxwell model cannot describe properly the variation in electrical conductivity when nanoparticles are added to the base fluid (i.e., it under predicts the experimental values), as well as other classical theoretical models.

7. Only few equations for estimating electrical conductivity are present in the open literature, most being linear correlations.

8. None of the reviewed studies discussed about the preferred application of manufactured nanofluids, based on their electrical conductivity performance.

As a general conclusion, it was noticed that even if the research on nanofluids started a couple of decades ago, the majority of electrical conductivity studies are limited to nanofluids based on water, EG and few W-EG mixtures. Other base fluids studies are scattered and a solid conclusion cannot be ruled out yet. Another observation, this time in regard to ionic liquid-enhanced nanofluids (NEIL), is that no studies are available to date in the open literature in regard to their electrical behaviour, even if the manufacture of these NEILs was firstly noticed about 8 years ago.

### Challenges and Future Directions for Research

In spite of their superior characteristics, nanofluids are still under-developed at this moment for most industrial applications and a better characterization, especially in regard to practical or preferred real-life applications, might be a great plus for nanofluid technology’s readiness level increase. Summarizing, the challenges, correlated with the future directions for research can be identified as:

1. Coordinated research is needed to check the appropriateness for evaluating electrical conductivity as an indicator of nanofluid stability.

2. A coordinated study of the overall electrical properties, including the electric conductivity. As an overall conclusion of the state of the art one may notice an increase of the electrical conductivity with nanoparticle concentration and temperature. Consequently, it is very important to intensify the research on electrical conductivity, especially in regard to different influencing factors such as the base fluid type (for example: polar or non-polar one), nanoparticle type and size, surfactant use and concentration effect. 

3. Another challenging point may be to obtain some valid correlations to describe the overall electrical conductivity enhancement.

4. Further consideration is also required to study the significance of nanofluid usage on lifetime improvement of thermo-electrical systems.

5. Another aspect to be considered, that even if very important is less studied, is the overall economics of regular fluids replacement by nanofluids. In future, the cost efficiency of nanofluids has to be a relevant direction to be addressed in detail.

Concluding, despite the availability of many potential applications, to date there are few to no reported industrial applications that involve nanofluids. With increasing research, it is expected that nanofluids can make a substantial impact as heat-transfer fluids in many applications (such as, for example, electronic cooling, automotive industry, and solar energy).

## Figures and Tables

**Table 1:** Outline of experimental work on electrical conductivity of nanofluids.

Base Fluid	Nanoparticle Type	Observation	Relevance as a Conductive Fluid, if Compared to the Base Fluid	Equipment Used for Electrical Conductivity Measurement	Reference
ethylene glycol (EG)	nitrogen doped activated carbon/graphene (NACG)	Increase was noticed while the samples concentration increases.	yes	AB200, Fisher scientific	Shirazi et al. [22]
	MgO and Si-TiO	Theoretical study using an artificial neural network (ANN) model.	no information provided	–	Mohamed [23]
	β-SiC	The variation with volume concentration was found to be linear.	yes	SG 23 SevenGo Duo, Mettler Toledo	Akilu et al. [24]
	In_2_O_3_	Maximum growth in electrical conductivity of In2O3–EG nanofluids was detected for 0.0081% concentration at 333.15 K, 27,300%.	yes	MultiLine 363	Fal et al. [25]
	graphene	Enhancement up to 220%.	yes	not declared	Baby and Ramaprabhu [26]
	nanodiamond	Maximum electrical conductivity enhancement was for 0.0338 volume fraction of nanoparticles (98 times higher than EG).	yes	Multiline 3630 (WTW GmbH, Weilheim, Germany)	Zyla et al. [27]
	aluminum nitride	Increase of up to 600 times in electrical conductivity with the upsurge in nanoparticles concentration.	yes	MultiLine 3410	Zyla and Fal [28]
	SiO_2_	Thermo-electrical conductivity (TEC) analysis revealed that there are no benefits from using this nanofluid in heat transfer processes.	yes	MultiLine 3410	Zyla and Fal [29]
	silicon oxide lignin (SiO_2_-L)	Increasing mass fraction, the conductivity increases.	yes	MultiLine 3410	Fal et al. [30]
	Si_3_N_4_	High progression in electrical conductivity due to several factors and especially due to concentration increase.	yes	MultiLine 3630m	Zyla et al. [31]
water	Al_2_O_3_	Highest value of electrical conductivity, 2370 µS/cm, was logged for 0.2% concentration at a temperature of 25.9 °C.	yes	not declared	Zawrah et al. [32]
	Fe_3_O_4_	A considerable enhancement of electrical conductivity with the upsurge in concentration and temperature.	yes	WagtechEc-meter model Con 11	Bagheli et al. [33]
	CuO	Enhancement of electrical conductivity with the increase in temperature and volume concentration.	yes	EC-Meter GLP 31 from CRISON	Coelho et al. [34]
	un-doped and zinc doped cobalt ferrite	Maxwell model is not suitable.	no information provided	Cyberscan CON110	Anu and Hemalatha [35]
	Alumina CuO MgO CNT titania ZnO	Linear correlations were proposed by authors.	yes	JENWAY 4520	Shoghl et al. [36]
	Ag SiC Graphene oxide (GO)	Electrical conductivities of nanofluids is linearly increasing with temperature and concentration.	yes	AZ86505 benchtop multi-meter	Heyhat and Irannezhad [37]
	Fe_2_O_3_	Enhancement of electrical conductivity with the increase in temperature and volume concentration.	yes	Eutech instrument PC 2700	Nurdin and Satriananda [38]
	diamond	Electrical conductivity was found lower than similar concentrations of other nanoparticles.	no	Orion A122 Conductivity Meter (Thermo-Orion, Boston, USA)	Mashali et al. [39]
	TiO_2_	Enhancement in electrical conductivity in dependence with nanoparticle addition.	yes	digital conductivity meter (Dip cell, Pt plate surface, Model 1054, Amber Science Inc., OR, US)	Modesto-Lopez and Biswas [40]
	graphene	Enhancement up to 1400%.	yes	–	Baby and Ramaprabhu [26]
	Al_2_O_3_	An increase in electrical conductivity of about 5.5. times compared to water.	yes	CyberScan PC10	Zakaria et al. [41]
	graphene oxide (GO)	Enhancement of electrical conductivity.	yes	BA 380	Hadadian et al. [42]
	Al_2_O_3_	A disagreement was noticed between diverse electrical conductivity models for upper values of Richardson number.	no information provided	–	Selimefendigil and Öztop [43]
	Al_2_O_3_	Considerable augmentation of electrical conductivity with volume fraction.	yes	Tetracon	Ganguly et al. [44]
	Al_2_O_3_	At room temperature an increase of 379.6% in effective electrical conductivity of nanofluid is detected for 4% alumina.	yes	Multiparameter Consort C 831	Minea and Luciu [45] Minea [46]
	ND-Ni nano-composite	A disagreement was noticed between experimental results and conventional models.	no information provided	two-pole conductivity electrode meter (Mettler-Toledo, USA)	Sundar et al. [47]
	TiO_2_ SiO_2_ Alumina + titania hybrid alumina + silica hybrid	Large enhancement of electrical conductivity was noticed, depending also on the nanoparticles synergy.	yes	Edge® Multiparameter HI 2030 (Hanna Instruments)	Chereches and Minea [48]
EG–water mixture	TiO_2_	Experimental results point out that the Maxwell model is not capable to foretell the electrical conductivity.	yes	IntelliCALTM CDC401	Islam et al. [49] Islam and Shabani [50]
	Cu Al_2_O_3_ CuO	The Maxwell model under predicts the experiment.	yes	CYBERSCAN CON 11	Sarojini et al. [51]
	SiO_2_	The electrical conductivity rises by about 10 times.	yes	Jenco Instruments Inc	Guo et al. [52]
	graphene	Electrical conductivity rapidly increased with loading of GONs until 0.07 wt.%.	depending on concentration	Orion™ VERSA STAR™ Multiparameter Benchtop Meter	Ijam et al. [53]
	functionalized graphene nanosheets	Electrical conductivity enhanced to a percentage up to 8620%.	yes	–	Kole and Dey [54]
	Al_2_O_3_	The change in electrical conductivity is rather low.	no	CyberScan PC10	Zakaria et al. [41]
bio glycol (BG)	Al_2_O_3_	Electrical conductivity increases with temperature.	yes, even if the alumina addition decreases slightly the electrical conductivity of BG	Cyberscan PC-10	Khdher et al. [55]
bio glycol–water mixture	Al_2_O_3_	Electrical conductivity of BG:W in 40%:60% decreased progressively while adding nanoparticles.	no	Cyberscan PC-10	Abdolbaqi et al. [56]
diesel oil	Graphene multi-wall carbon nanotubes (MWCNT)	Nanofluids with functional nanomaterials have inferior electrical conductivity compared to those with non-functional ones.	no, because Diesel has extremely low electrical conductivity	non declared electrical property analyzer	Naddaf and Heris [57]

**Table 2:** Summary of several experimental work on electrical conductivity of base fluids.

Base Fluid	Electrical Conductivity (µS/cm)	Reference
EG	0.12	Akilu et al. [24]
PG	0.10	
Distilled water	6	Zakaria et al. [41]
EG	1.07	
EG	3.14	Islam et al. [49]
EG-Water 50:50	5.03	
Water	5.44	Guo et al. [52]
EG-Water 20:80	4.22	
EG-Water 40:60	1.9	
EG-Water 60:40	1.47	
EG-Water 80:20	1.36	
EG	0.33	
Distilled water-EG 60:40	12.7	Ijam et al. [53]
Distilled water	6	Abdolbaqi et al. [56]
BG	45	
BG	53	Khdher et al. [55]
BG-Water 60:40	389	Abdolbaqi et al. [56]
BG-Water 40:60	620	
Diesel oil	authors cannot measure it	Naddaf and Heris [57]

**Table 3:** Outline of experimental work on electrical conductivity variation with temperature.

Base Fluid	Nanoparticle Type	Temperature Influence over Electrical Conductivities Values	Reference
EG	nitrogen doped activated carbon/graphene (NACG)	• maximum enhancement of 11,000% at 30 °C for 0.06%. temperature does not linearly influence the electrical conductivity values, a decrease was noticed at 35 °C and no explanation was provided	Shirazi et al. [22]
	β-SiC	• maximum enhancement of 53.5% for 1 vol.% the dependence of β-SiC NFs electrical conductivity on temperature can be modelled using an Arrhenius-type equation	Akilu et al. [24]
	In_2_O_3_	• the highest increase in electrical conductivity was achieved for 0.0081 vol.% at temperature of 333.15 K and it was 272 times higher than that in case of pure ethylene glycol at 298.15 K.	Fal et al. [25]
water	Fe_3_O_4_	• maximum enhancement of 360% at 65 °C	Bagheli et al. [33]
	CuO	• The conductivity increases with increasing temperature	Coelho et al. [34]
	un-doped and zinc doped cobalt ferrite	• up to 94% enhancement at 308 K the percentage enhancement in electrical conductivity decreases with the increase in temperature, as thermal agitation hinders the percolation behavior	Anu and Hemalatha [35]
	Ag SiC Graphene oxide (GO)	• the maximum augmentation occurred in temperature of 25 °C and weight fraction of 0.05% GO maximum enhancement of 15 times higher at 50 °C for 1% SiC	Heyhat and Irannezhad [37]
	Fe_2_O_3_	• up to 22% enhancement for 2.5% at 60 °C	Nurdin and Satriananda [38]
	Al_2_O_3_	• up to 115% for 3% at 45 °C	Ganguly et al. [44]
	Al_2_O_3_	• linear increase with temperature	Minea and Luciu [45]
	ND-Ni nano-composite	• linear increase with temperature maximum electrical conductivity enhancement for 0.1% ND–Ni is 1339.81% at 24 °C	Sundar et al. [47]
	TiO_2_ SiO_2_ alumina + titania hybrid alumina + silica hybrid	• linear increase with temperature	Chereches and Minea [48]
EG-water mixture	TiO_2_	• maximum enhancement of 13 times higher for 0.5% at 70 °C	Islam et al. [49] Islam and Shabani [50]
	Cu Al_2_O_3_ CuO	• linear increase of electrical conductivity with temperature. no appreciable improvement of electrical conductivity with temperature for low volume fractions (less than 0.5%)	Sarojini et al. [51]
	SiO_2_	• up to 10 times at 45 °C for the nanofluid with water	Guo et al. [52]
	graphene	• at 25 °C, maximum improvement in electrical conductivity is 1664% at 0.10% concentration	Ijam et al. [53]
bio glycol	Al_2_O_3_	• maximum enhancement of 5112% was obtained by 0.1% Al_2_O_3_ at 30 °C temperature linearly influences electrical conductivity	Khdher et al. [55]
diesel oil	Graphene MWCNT	• temperature linearly influences electrical conductivity in the range 5–100 °C	Naddaf and Heris [57]

## Abbreviations

**Nomenclature**	
*K_0_*	fitting parameter in Hill equation, -
L	Avogadro constant, L = 6.02214086 × 10^23^ mol^−1^
*n*	fitting parameter in Hill equation, -
*r*	radius of nanoparticles, nm
R	thermodynamic constant, R = 8.314 kJ/kmol K
*t*	temperature, °C
*T*	temperature, K
*U_0_*	zeta potential, mV
**Greek Symbols**	
*α*	ratio of electrical conductivity, -
*ε_0_*	dielectric constant of vacuum, -
*ε_r_*	dielectric constant of base fluid, -
*φ*	volume fraction of nanoparticles, -
*η*	viscosity, Pa.s
*ρ*	density, kg/m^3^
*σ*	electrical conductivity, µS/cm
**Subscripts**	
*B*	refers to Brownian motion
*bf*	refers to base-fluid
*E*	refers to electrophoretic mobility
*M*	refers to Maxwell model
*nf*	refers to nanofluid
*p*	refers to nanoparticles
**Abbreviations**	
ANN	artificial neural network model
BG	Bio glycol
CNT	carbon nanotubes
EDL	electrical double layer
EHD	electro hydrodynamics
EG	ethylene glycol
MHD	magneto hydrodynamics
MWCNT	multi-wall carbon nanotubes
NEIL	ionic liquids enhanced nanofluids
PG	propylene glycol
RDB	refined, bleached and deodorized (refers to oils)
SDBS	Sodium dodecyl benzenesulfonate
SDS	Sodium dodecyl sulfate
TEC	thermo-electrical conductivity
W	water
